# A shared DNA-repeat toxicity threshold, reached somatically at cell-type-specific rates, unites cortical and striatal neurodegeneration in Huntington’s disease

**DOI:** 10.64898/2025.12.09.688862

**Published:** 2025-12-11

**Authors:** Seva Kashin, Won-Seok Lee, Tara M. McDonald, Kiely Morris, Robert E. Handsaker, Curtis Mello, Liv Spina, Nora M. Reed, Heather de Rivera, Sri Havya Jana, Marina Hogan, Sabina Berretta, Steven A. McCarroll

**Affiliations:** 1. Broad Institute of MIT and Harvard, Cambridge, MA 02142, USA; 2. Department of Genetics, Harvard Medical School, Boston, MA 02115, USA; 3. McLean Hospital, Belmont, MA 02478, USA; 4. Department of Psychiatry, Harvard Medical School, Boston, MA 02215, USA; 5. Program in Neuroscience, Harvard Medical School, Boston, MA 02215, USA; 6. Howard Hughes Medical Institute, Boston, MA 02215, USA

## Abstract

Huntington's disease (HD) affects two major brain areas – the striatum and cerebral cortex – in ways that differ in timing, severity, and gene-expression changes. For these reasons, and because many cortical neurons project axons to the affected striatal neurons, striatal and cortical atrophy have long been proposed to have distinct mechanisms, with one potentially a secondary consequence of the other.

In the striatum, we recently found that neurons degenerate asynchronously as their own huntingtin (*HTT)* gene CAG-repeat tracts, typically inherited at 40-50 CAGs, expand somatically beyond 150 CAGs. To ask whether a similar or different dynamic affects the cerebral cortex, we analyzed *HTT* CAG repeats and genome-wide RNA expression together in more than 130,000 nuclei from 12 cortical areas of brain donors with HD.

The resulting data revealed that cortical and striatal neurodegeneration in fact result from analogous sequences of cell-autonomous events, each instructed by somatic expansion of a neuron's own *HTT* CAG repeat. Analyses revealed that somatic expansion beyond a high toxicity threshold (of about 150 CAGs) is necessary and sufficient to initiate pathological changes; that this pathogenicity length threshold is shared by striatal and cortical projection neurons of all types; and that cortical area, cortical layer, and axonal projections play only incidental roles, as proxies for the true driver: profound (up to 50-fold) variation among types and subtypes of pyramidal neurons in the likelihood of reaching the 150-CAG toxicity threshold in a human lifetime. These results also suggest that containing somatic DNA-repeat expansion below this high toxicity threshold would protect both brain areas in HD.

## Introduction

Huntington's disease (HD) is a neurodegenerative disease involving progressive loss of neurons in the striatum and cerebral cortex, leading to escalating motor, cognitive and psychiatric impairments. The most prominent disease-associated change is atrophy of the striatum (caudate and putamen) due to loss of striatal projection neurons (SPNs, also known as medium spiny neurons or MSNs). HD also affects the cerebral cortex, causing loss of pyramidal projection neurons, especially in deep cortical layers ^1-5^, which harbor a variety of pyramidal neuron types, including neurons that project axons corticostriatally to SPNs.

Striatal and cortical pathology in HD appear different in many ways. In the striatum, HD causes loss of inhibitory (GABAergic) SPNs; in the cerebral cortex, HD causes loss of excitatory (glutamatergic) pyramidal neurons ^5^. While most to all SPNs are lost in advanced HD, only a minority of cortical pyramidal neurons are lost. Profiling gene expression in striatal and cortical neurons in HD patients and HD mouse models has not identified strong correlations between the gene-expression changes in the two brain areas ^6-8^, and studies suggest distinct mechanisms for the two ^6-8^. These and earlier observations have long been interpreted to suggest that striatal and cortical pathology in HD may have different mechanisms and especially that, due to corticostriatal projections, pathology in one brain area elicits secondary pathology in the other brain area – for example, due to excitotoxicity ^9-11^, corticostriatal disconnection ^6,7,12,13^, or spreading proteins with prion-like properties ^14,15^.

HD is caused in a genetically dominant manner by an inherited expansion of a DNA-repeat tract ([CAG]_n_) in the first exon of the huntingtin (*HTT*) gene. This inherited CAG-repeat tract – which is usually 15 to 30 CAGs long – has been inherited at 36 or more CAGs (most frequently 40-50) in all persons with HD ^16^. Age at onset of clinical HD symptoms is a more complex trait: in addition to being shaped by CAG-repeat length (longer inherited CAG-repeat tracts associate with earlier onset), age at clinical motor symptom onset is also shaped by common genetic variants at many loci. Several of the implicated loci contain genes that affect the stability of DNA repeats ^17-29^. This constellation of human genetic and biological results has suggested that somatic instability may contribute to HD trajectories.

We recently described experimental results supporting a model for striatal HD pathology, in which long somatic expansions of this inherited CAG repeat tract, attained via many incremental length increases over decades, lead to the loss of striatal projection neurons (SPNs) ^30^. First, the CAG-repeat tracts in SPNs expand somatically over decades via many incremental length-change mutations (phase A). Upon expanding beyond about 90 CAGs, a tract's expansion accelerates, reaching 150 CAGs in just a few years (phase B). When an SPN's CAG-repeat tract expands beyond about 150 CAGs, the cell begins to exhibit repeat-length-associated changes in gene expression (phase C); these changes progressively increase in magnitude and affect expression of hundreds of genes. Individual SPNs later enter a "de-repression crisis" (phase D) in which they de-repress scores of genes that are normally silent. These SPNs are soon lost (phase E).

It is not known whether such a dynamic can also explain cortical neurodegeneration in HD. Key challenges for such a model would be to explain the many ways in which cortical and striatal neurodegeneration differ – including in timing, severity, cell-type specificity, and gene-expression changes.

To address these and other questions, we applied an experimental approach we recently invented for analyzing *HTT* CAG-repeat length and genome-wide RNA expression together at single-cell resolution ^30^. We analyzed more than 100 thousand cell nuclei sampled *post mortem* from the cerebral cortex of eight persons with HD, 15 times more nuclei than in our previous work on striatum.

We find that cortical and striatal neurodegeneration actually do arise from analogous sequences of cell-autonomous events, each instructed by somatic expansion of the *HTT* CAG repeat; that somatic expansion beyond a high toxicity threshold is necessary and sufficient to initiate these events; that this length threshold is shared by striatal and cortical projection neurons of all types; and that long-studied variables, such as cortical area, laminar locations, and axonal projections play only minor or incidental roles as proxies for what truly matters: a profound effect of a neuron's precise molecular identity on its somatic expansion kinetics, which determine the likelihood that the high toxicity threshold is reached in a human lifetime.

## Results

### Long repeat expansions, beyond 100 CAGs, in glutamatergic neurons

In the striatum, HD causes loss of most SPNs while sparing other cells. This cell-type specificity corresponds to the cell-type specificity of long somatic expansions (beyond 150 CAGs), which manifest almost exclusively in SPNs ^30^. (Cholinergic interneurons exhibit moderate somatic expansion ^6^ but relatively few of them reach the high toxicity threshold of ~150 CAGs, while large fractions of SPNs do so ^30^.)

In the cerebral cortex in HD, the primary degenerating cells are pyramidal projection neurons ^2-4^, which are excitatory (glutamatergic) and differ markedly in gene expression and physiology from SPNs, which are inhibitory GABAergic neurons.

To assess the cell-type specificity of somatic expansion in the cerebral cortex, we measured CAG-repeat length and genome-wide RNA expression together at single-cell resolution, using a laboratory approach we recently developed ^30^. We generated such paired measurements for 78,095 nuclei sampled from 12 cortical areas (these areas and their functions are elaborated in Suppl. Table 1) of a 48yo person with HD who had inherited a CAG-repeat tract of 42 CAGs. We also analyzed 60,482 nuclei sampled from seven additional persons with HD, focusing on motor cortex (Brodmann Area 4, BA4) and anterior cingulate cortex (Brodmann Area 32, BA32).

As in the striatum, somatic expansion appeared to be allele-specific, affecting the HD-causing allele but not the other allele (Suppl. Fig. 1).

**Figure 1.**
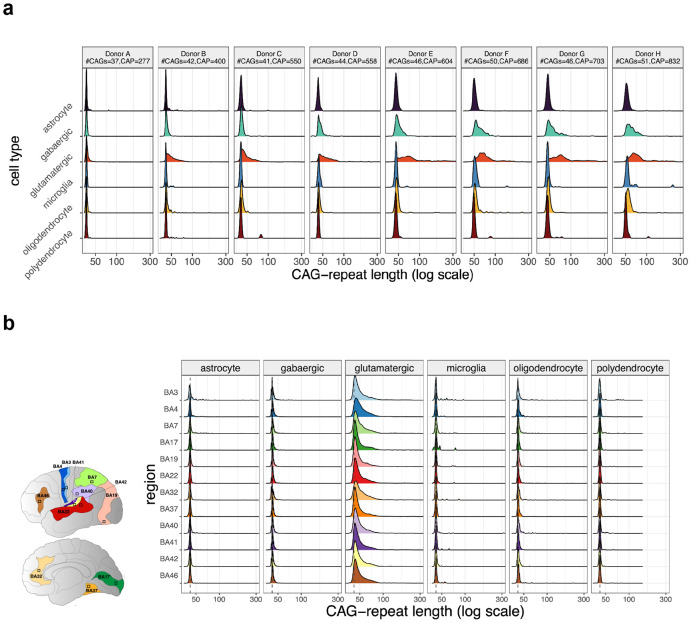
Cell-type-specific variation in somatic CAG-repeat expansion in the cerebral cortex. In each analysis, brain tissue was analyzed by a single-cell-resolution laboratory approach that allows joint measurement of genome-wide RNA expression and *HTT* CAG-repeat sequence. Genome-wide expression data were used to assign each CAG-repeat sequence to the cell type from which it was derived. (**a**) Analysis of motor cortex (Brodmann area 4) from eight brain donors with HD, ordered (from left to right) based on increasing CAP score, an estimate of HD prognosis/progression based on age and inherited CAG-repeat length. (**b**) Analysis of twelve cortical areas in one of the above donors (donor B; BA3, primary somatosensory; BA4, primary motor; BA7, multisensory visual/somatosensory associative; BA17, primary visual; BA19, visual associative; BA22, auditory associative; BA32, anterior cingulate; BA37, visual high level associative; BA40, somatosensory associative; BA41, primary auditory; BA42, auditory associative; BA46, dorsolateral prefrontal, executive functions). Density plots show CAG-repeat length distributions for six cortical cell types (columns) in 12 cortical areas (rows). While panels **a** and **b** focus on the HD-causing allele, Suppl. Fig. 1 contains data for both alleles, allowing visualization of the allele specificity of the repeat expansion.

The HD-causing allele exhibited some CAG-repeat instability in all cell types, but by far the most expansion in glutamatergic (pyramidal) neurons (Fig. 1a), which were the only cortical cell type for which an appreciable fraction of cells had expanded their CAG-repeat tracts beyond 100 CAGs at the end of life. This pattern replicated strongly across all cortical areas (Fig. 1b). (Cortical GABAergic neurons exhibited the next most somatic expansion – mainly in donors who had inherited CAG-repeat tracts longer than 45 CAGs – but even in those donors, only a tiny fraction of such neurons reached 100 CAGs (Fig. 1a and Suppl. Fig. 2).)

Thus, long somatic expansions – exceeding 100 CAGs in length – arise among the cortical cells that are lost in large numbers in HD (Fig. 1), and only from the HD-causing allele, analogous to the pattern described in the striatum ^30^.

### Somatic expansion varies profoundly across pyramidal neuron types

Though we detected long somatic CAG-repeat expansions (beyond 100 CAGs) in only about 1-2% of pyramidal neurons, such expansions could in principle be arising primarily in specific types of pyramidal neurons, and thus affect larger fractions of those neurons.

Historically, knowledge about the vulnerabilities of cortical neurons in HD has come mostly from studies based on neuroimaging, which recognizes volume and activity changes in cortical areas, and from microscopy and stereological counting, which also recognize the laminar locations of neurons. These studies have found that HD pathology occurs across cortical areas ^1^ and that, while all cortical layers exhibit some neuronal loss, deeper cortical layers (5 and 6) exhibit more neuronal loss than upper layers (2,3,4) do ^4^.

Cortical pyramidal neurons are increasingly classified, based on RNA-expression patterns, into ever-more-elaborate "molecular" taxonomies of types and subtypes at increasing levels of resolution ^31-34^. The early scRNA-seq taxonomies (e.g. refs^31,33^) have been annotated with anatomic information on laminar locations and axonal projections; the most recent taxonomy ^32^ has the most fine-grained subdivisions (but less annotation).

**Figure 2.**
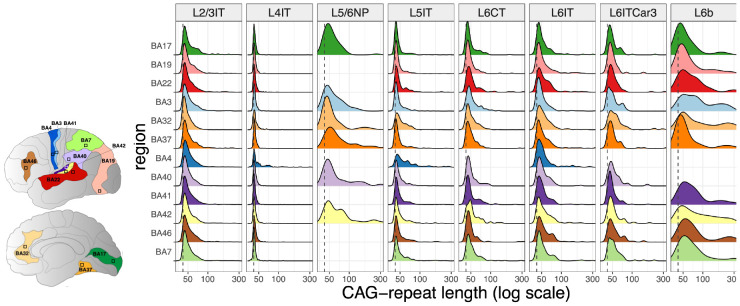
Analysis of somatic CAG-repeat expansion in 47,979 pyramidal neurons sampled from 12 cortical areas of a person with HD. (**a**) Twelve cortical areas sampled. These areas and their functions are elaborated in Suppl. Table 1. (**b**) Each cortical area harbors pyramidal neurons with a variety of molecular identities or types; these types of pyramidal neurons exhibit considerable variation in the amount of somatic expansion realized during a human lifetime. (Note that we included curves only for those region/celltype combinations for which we obtained measurements for at least 20 cells.)

We first used the Hodge taxonomy ^31^ as a scaffold for analysis of somatic expansion in eight major pyramidal neuron types (those that were sufficiently well-represented for statistically well-powered analysis in a cortical area) (Fig. 2). Within any one cortical area, these types of pyramidal neurons varied greatly in the amounts of somatic CAG-repeat expansion that they had acquired over a lifetime (Fig. 2). For example, in the anterior cingulate cortex (BA32), 43% of layer 6b (L6b) neurons had expanded their CAG-repeat tract beyond 100 CAGs, but only 0.5% and 0.9% of layer 4 and layer 2-3 intratelencephalic-projecting neurons (L4IT and L2/3IT, respectively) had done so (Fig. 2).

The pyramidal neuron types that were high-expansion or low-expansion in one cortical area tended to have this same property in all cortical areas analyzed (Fig. 2). For example, layer 6b (L6b) pyramidal neurons (last column in Fig. 2) were the most repeat-expanded pyramidal type in all cortical areas in which they were sufficiently numerous for conclusive analysis, whereas layer 4 intratelencephalic-projecting (L4IT) neurons (second column in Fig. 2) were the least repeat-expanded type in all cortical areas.

These results reveal that somatic expansion rates are persistent features of pyramidal neurons' molecular identities.

Across cortical areas, two types of pyramidal neurons – layer 6b (L6b) and layer 5/6 near-projecting (L5/6NP) neurons – exhibited far more somatic CAG-repeat expansion than other neurons did (Fig. 2).

Notably, high-somatic-expansion pyramidal types (L6b, L5/6NP) were characterized not only by high mean expansion but by high variance (in expansion) across individual cells of the same type (Fig. 2), almost certainly reflecting the way that somatic expansion events are stochastic (random) and initially infrequent, but expansion increases the likelihood of subsequent expansion.

The axonal projections of pyramidal-neuronal types have been systematically characterized by retrograde labeling experiments in mice ^33^. Such experiments have found that L6b neurons project intracortically and to the thalamus, and that L5/6NP neurons project intracortically. Corticostriatal projections have been found among L5IT and L6IT neurons ^33^, which exhibited just modest somatic expansion in our analysis (Fig. 2).

The above results (Fig. 2) had suggested a strong effect of a pyramidal neuron's precise molecular identity, relative to the effect of cortical area. To rigorously measure such effects, and to expand analysis to a more fine-grained set of pyramidal-neuron molecular identities (subtypes), we performed negative binomial regression analysis, regressing each individual neurons' CAG-repeat tract length against independent variables for cortical area and pyramidal neuron subtype. The large number of neurons (~80 thousand) in this pan-cortical analysis provided sufficient statistical power to analyze the larger number of fine-grained pyramidal-neuron molecular identities (subtypes or "cluster names" in ^32^) in the Siletti taxonomy. (We also mapped each of the Siletti clusters to its corresponding supertype (L2/3IT, L5/6NP, etc.) in the earlier Hodge taxonomy by matching RNA-expression patterns (see Methods), to benefit from earlier spatial and connectivity characterizations of the the Hodge taxonomy.)

**Figure 3.**
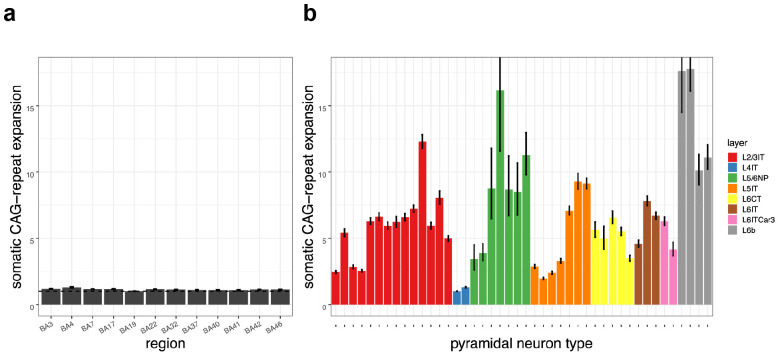
Effects of cortical area/region, molecular identity (pyramidal neuron type), and laminar location (layer) upon cortical glutamatergic neurons' somatic CAG-repeat expansion. The heights of the bars represent the exponentials of the model coefficients from a regression model (fitted to data from 47,479 neurons) in which somatic expansion is explained as a function of a neuron's cortical area (**a**) and molecular identity (**b**). The 44 molecular identities in (**b**) correspond to clusters in the Siletti taxonomy, each of which we also connected (via RNA expression patterns) to its corresponding supertype (L2/3IT, L5/6NP, etc., represented by colors) in the earlier Hodge taxonomy.

We first analyzed 47,479 neuronal nuclei sampled from 12 cortical areas of a single donor. Analysis revealed that cortical area had at most a modest effect on a neuron's propensity for repeat expansion, with cortical areas varying by only up to 1.3-fold in somatic expansion when controlling for the molecular identities of the neurons they contained (Fig. 3a).

In contrast to the modest effect of cortical area, molecularly defined pyramidal subtype had a profound effect, with pyramidal subtypes exhibiting up to 18-fold variation in the amounts of somatic expansion their CAG-repeat tracts had undergone (Fig. 3b).

To assess whether these relative expansion rates were general (as opposed to donor-specific) features of these neuronal subtypes, we also analyzed 33,430 neuronal nuclei sampled from seven additional brain donors with HD, in whom we sampled motor cortex (BA4) and anterior cingulate cortex (BA32) (13,760 neuronal nuclei from BA4; 19,670 from BA32). The resulting estimates of the glutamatergic neuronal types' relative expansion propensities were highly correlated across these analyses, regardless of the donor(s) or cortical area(s) in which they were measured (Suppl. Fig. 3), suggesting that somatic-expansion rates are persistent features of pyramidal neurons' precise molecular identities.

The laminar locations of molecularly defined pyramidal-neuron types have been characterized by earlier spatial transcriptomics analyses ^34^, making it possible to ask about the relationship of somatic expansion to cortical layer. The pyramidal types with the most somatic CAG-repeat expansion (L6b, L5/6NP) are among the types found in deep layers 5 and 6, potentially aligning with longstanding observations that HD pathology affects deep layers earlier and more severely than it affects upper layers ^4^. However, all cortical layers contain mixtures of pyramidal neuron subtypes that our analyses found to have a variety of expansion rates. For example, upper-layer (L2/3IT) projection neurons include L2/3IT subtypes with diverse amounts of expansion (red bars in Fig. 3b, see also Suppl. Fig. 4), and deep-layer L5/6NP neurons include L5/6NP subtypes (shortest green bars in Fig. 3b, see also Suppl. Fig. 4) with very little somatic expansion.

Our experimental results indicate that molecularly defined types and subtypes of cortical projection neurons have intrinsic propensities for somatic expansion that are consistent across cortical areas, consistent across people, that correlate only partially with laminar locations, and that do not distinguish cortistriatal projection neurons. Somatic expansion could in principle be better explained by one or more features of pyramidal neurons' fine-grained molecular identities, such as activity patterns or metabolic properties, which generally have yet to be characterized ^35^. We tested one simple possibility – that the nuclear RNA expression levels of several DNA-repair genes might substantially explain this variation. Although these genes' nuclear RNA expression levels exhibited modest variation (generally less than two-fold) across pyramidal neuron types, this variation appeared not to be nearly large enough to explain the 18-fold variation in these same neuronal types' somatic DNA-repeat expansion (Suppl. Fig. 5).

### Somatic expansion in two phases (A and B), as in SPNs

In the striatum, SPN CAG-repeat lengths exhibit a mathematical distribution that visually resembles the profile of an armadillo, with a large majority of neurons having expanded to 45-90 CAGs (the armadillo's body), and a small fraction having attained far-higher CAG-repeat lengths of 100 to 500+ CAGs (the tail) ^30^. These distributions are likely to arise from a two-stage somatic-expansion process in which somatic expansion is initially slow (phase A, requiring decades to expand from 40 to 90 CAGs) but accelerates – beyond its general pattern of continuous increase with repeat length – after expansion past about 90 CAGs (phase B) ^30^.

CAG-repeat-length distributions in cortical projection neurons of all types also exhibited armadillo-like shapes (Suppl. Fig. 6), suggesting that this two-phase expansion regime is a shared property of striatal and cortical projection neurons.

### Phase C: gene expression pathology commences at about 150 CAGs

The key question for understanding HD pathology in the cerebral cortex involves whether, when and how the length of the disease-causing CAG repeat affects the biology of cortical neurons. We thus sought to recognize relationships between the length of this CAG-repeat tract in individual neurons and the same neurons' patterns of gene expression. We performed negative binomial regression analysis of each gene's expression levels (across all the individual neurons in the analysis), controlling for the effects of fine-grained molecular identities (of the neurons) and of donor-specific effects by including molecular identity and donor as additional independent variables in the analysis. This analysis identified more than 400 genes whose expression levels correlated with the lengths of glutamatergic neurons' CAG-repeat tracts.

**Figure 4.**
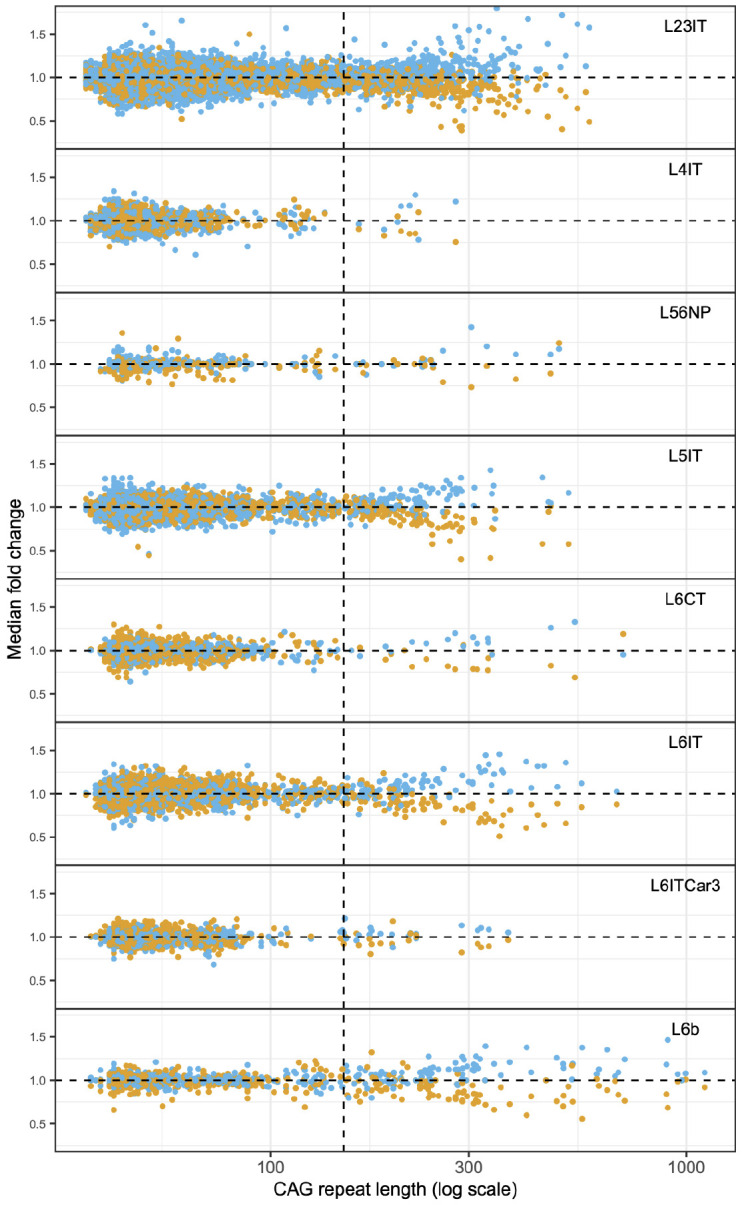
Gene-expression changes in cortical neurons with somatic CAG-repeat expansion. Each of the eight plots shows the data for a specific type of cortical glutamatergic neuron. On each plot, each neuron is represented by both a blue point and an orange point: orange points show the median fold-change of a set of 115 genes that decrease in expression with CAG-repeat expansion (C- genes); blue points show the median fold-change of a set of 142 genes that increase in expression with CAG-repeat expansion (C+ genes). The same genes are used for the calculation in each panel. Suppl. Fig. 6,7 contain data from additional brain donors with HD.

These gene-expression changes were apparent in those pyramidal neurons that had expanded their CAG-repeat tracts beyond about 150 CAGs (Fig. 4; Suppl. Fig. 7,8). This was true regardless of a neuron's molecular identity, laminar location or reported connectivity (Fig. 4). The magnitude of these gene expression changes appeared to escalate alongside further CAG-repeat expansion (beyond 150 CAGs), similar to the trajectory in striatal neurons, though more modest in slope. (Neurons with shorter repeat expansions (less than 150 CAGs) exhibited a more-random pattern consistent with statistical sampling noise and/or biological fluctuations, Fig. 4.)

Importantly, these gene-expression changes had affected glutamatergic neurons *of all types*: at the single-neuron level, the critical variable involved was not molecular identity but rather the extent to which a neuron's CAG-repeat tract exceeded the 150-CAG threshold (Fig. 4, Suppl. Fig. 7,8). The effect of molecular identity was seen mainly in the fraction of neurons (of each type) that had crossed this threshold (Fig. 4; Suppl. Fig. 7,8).

**Figure 5.**
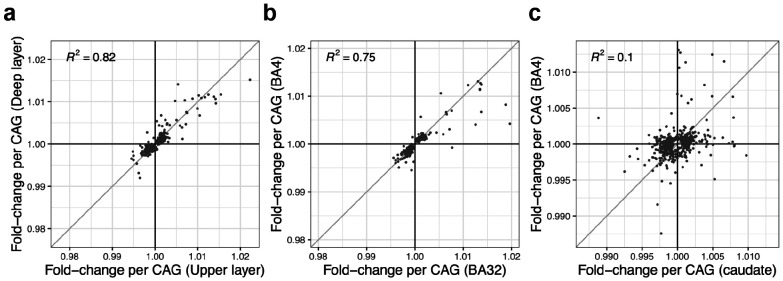
Comparisons of phase C gene-expression changes between (**a**) deep-layer vs. upper-layer cortical projection neurons, (**b**) cortical projection neurons in BA32 vs. BA4, and (**c**) cortical projection neurons vs. striatal projection neurons. Each point corresponds to a gene that exhibited significant (p < 10^−10^) evidence of phase C gene-expression change in at least one of the two sets of cells being compared. The quantities plotted on the x- and y-axes are the fold-changes per CAG-repeat unit expansion (beyond 150 CAGs), reflecting the tendency of these changes to escalate gradually and continuously alongside further CAG-repeat expansion.

The ensuing gene-expression changes (that commenced upon a neuron's crossing of the 150-CAG threshold) were also essentially identical across pyramidal types, for example when comparing upper-layer to deep-layer pyramidal neurons (Fig. 5a).

We call this period of continuously escalating transcriptional changes "phase C". Its progressive dynamic could reflect either intensifying toxicity (with further expansion) or the amount of time since the toxicity threshold was crossed.

These results indicate that, despite the long-noted differences between cortical and striatal pathology in HD, cortical and striatal projection neurons in fact share a common CAG-repeat length toxicity threshold.

### Phase C changes in cortical and striatal neurons are distinct

One of the mysteries that has long suggested the potential for distinct cellular mechanisms for cortical and striatal neuropathology in HD is that the pathological gene-expression changes in the two brain areas exhibit little reported correlation with each other ^6-8^.

Intriguingly, although phase C gene-expression changes in cortical neurons were highly similar across cortical areas and between deep-layer and upper-layer pyramidal neurons (Fig. 5ab), phase C changes in cortical neurons were quite different from phase C changes in striatal neurons (Fig. 5c). Thus, though striatal and cortical neurons share a similar CAG-repeat-length threshold for the commencement of these changes, phase C perturbs their gene expression (and thus potentially their physiologies) in quite different ways.

### Phase C changes in cortical and striatal neurons involve identity erosion

The hundreds of genes that decline in expression in SPNs during phase C share a specific property in common: almost all are genes whose expression distinguishes SPNs from other kinds of neurons ^30^ (see also analysis of a mouse HD model ^36^). Phase C in SPNs thus involves the steady erosion of positive features of SPN identity.

**Figure 6.**
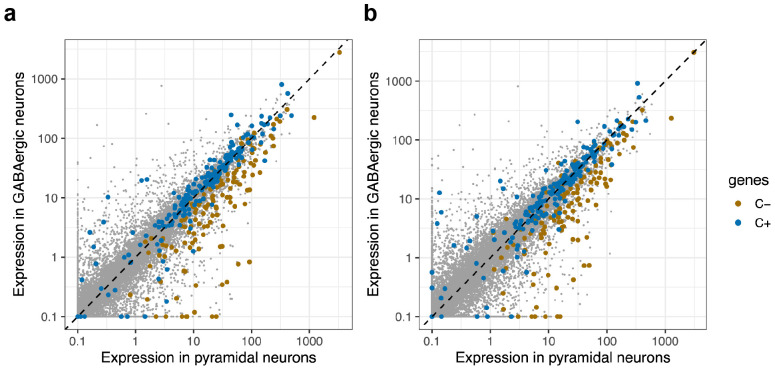
Genes that decline in expression during Phase C (C- genes) are genes that, under normal circumstances, are much more strongly expressed in cortical pyramidal neurons (CPNs) than in cortical interneurons. Gray points: all genes (cell-type-specific expression levels in unaffected individuals). Colored circles: Genes whose expression levels decline (orange) or increase (blue) in neurons with *HTT* CAG-repeat expansion beyond 150 units (in phase C). (**a**) motor cortex (BA4). (**b**) anterior cingulate cortex (BA32).

Intriguingly, the very different set of genes that declined in expression in cortical pyramidal neurons during their phase C exhibited this same property: the genes affected in pyramidal neurons were genes whose expression distinguishes pyramidal neurons from other neuronal types (Fig. 6). These genes encoded a wide variety of molecular functions. They included genes with categorical, on/off differences between pyramidal neurons and cortical interneurons – such as *SV2B*, *ENC1*, and *LMO4* – as well as genes with large quantitative differences in expression levels (higher in pyramidal neurons than interneurons), such as *PRKCB*, *NR3C1*, *KALRN*, and *ATP2B1*. However, phase C appeared to spare expression of many genes that distinguish types of pyramidal neurons from one another – such as *CUX2* (expressed in upper-layer pyramidal neurons), *FEZF2* (expressed in deep-layer subcerebrally projecting pyramidal neurons), and *THEMIS* (expressed in deep-layer intratelencephalically projecting neurons). (In fact, phase C gene-expression changes appeared to be shared almost identically between upper-layer and deep-layer pyramidal neurons, even when inferred in separate and independent analyses of these two groups of neurons (Fig. 5a). )

Thus, phase C gene-expression changes in striatal and cortical neurons are *conceptually* analogous and involve the same biological theme – positive features of the neurons' respective cell identities – while affecting different sets of genes.

### Cortical neurons undergo a de-repression crisis (phase D)

In the striatum, SPN pathology appears to culminate, after still-further somatic CAG-repeat expansion, in the de-repression of scores of genes that are normally silent in adult neurons ^30^. These genes include (i) HOX cluster genes, which are normally expressed only during embryonic development; (ii) a wide variety of transcription factors that are normally expressed in other neural cell types; and (iii) the *CDKN2A* and *CDKN2B* genes. This "phase D" appears to follow the onset of a cell's phase C by at least several months, as neurons with 150-250 CAGs have seldom entered phase D, whereas, with further repeat expansion (250+ CAGs) and progression of the phase C gene expression changes, ever-larger fractions of SPNs acquire this phase D de-repression phenotype ^30^.

**Figure 7.**
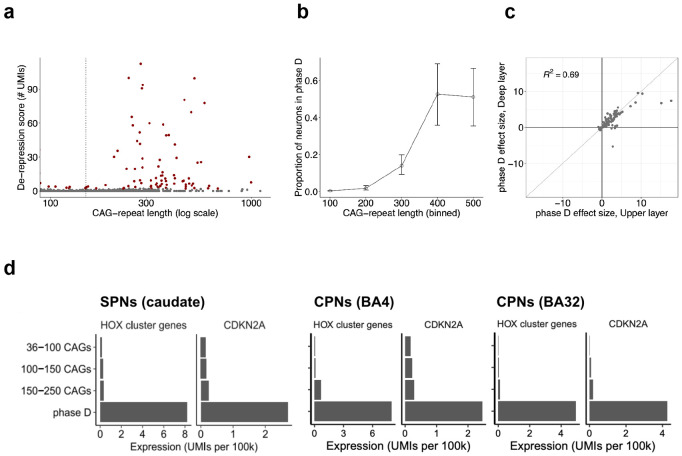
De-repression crisis (phase D). (**a**) De-repression of genes that are normally silent in cortical projection neurons. Points represent individual neuronal nuclei. y-axis: de-repression score, the number of transcripts (UMIs, normalized to 100k transcripts) detected from 59 phase D genes that are normally silent in cortical projection neurons. Replication analyses of data from additional person with HD are in Suppl. Fig. 8. (**b**) Proportion of cortical glutamatergic neurons that have entered phase D (y-axis), in relationship to the lengths of their *HTT* CAG-repeat tracts (x-axis), which is shown in bins. (**c**) Correlation of phase D gene-expression changes between upper-layer and deep-layer pyramidal neurons. Each circle point represents one gene; its location on the two axes corresponds to its log2-fold-change during phase D for either upper-layer pyramidal neurons (x-axis) or deep-layer pyramidal neurons (y-axis) as inferred from negative binomial regression analysis. Genes with significant phase D changes in at least one analysis are Note the preponderance of de-repression events (large positive fold-changes). (**d**) Expression of HOX cluster genes and *CDKN2A* in SPNs, cortical pyramidal neurons (CPNs) from BA4, and CPNs from BA32.

Cortical pyramidal neurons with particularly long CAG-repeat expansions also appeared to enter a de-repression crisis, expressing very many genes that are normally silent in neurons with CAG-repeat tracts shorter than 150 CAGs (Fig. 7a, Suppl. Fig. 8). As in SPNs, this "phase D" was seldom found among neurons with repeat tracts of 150-250 CAGs, but was found with increasing frequency among neurons with still-longer CAG-repeat tracts (Fig. 7b). Like phase C gene-expression changes, phase D gene-expression changes were shared almost identically between upper-layer and deep-layer pyramidal neurons (Fig. 7c)

As in SPNs, phase D resembled a discrete crisis that contrasted with the gradual, progressive changes of phase C: most neurons with >150 CAGs continued to have minimal expression of phase D genes (more than 85% of such neurons had only 0-1 unique RNA molecules (UMIs, normalized to 100k) detected across 59 phase D genes together). On the other hand, among neurons with more than 2 UMIs from phase D genes, more than 40% had at least 10 such UMIs and expressed up to 37 distinct phase D genes (Fig. 7a).

Moreover, unlike the phase C changes – whose magnitude was strongly correlated with a neuron's CAG-repeat length at the time of analysis (Fig. 4) – the length of a neuron's CAG-repeat tract did not associate with the magnitude of the phase D changes at the time of sampling (Fig. 7a). Rather, a neuron's CAG-repeat length associated with its probability of having entered phase D at all (Fig. 7b). All these results suggest that phase D involves a discrete crisis that cells enter rather suddenly, in contrast to the slowly, predictably escalating changes of phase C.

Phase D neurons de-repressed dozens of genes encoding transcription factors that are classic regulators of embryonic development. Even when selected using stringent criteria (FDR < 0.1%, fold-change > 10), more than half of the resulting 88 genes encoded transcription factors, including: 15 HOX cluster genes (Fig. 7d), which are normally involved in embryonic patterning; PAX family transcription factors (PAX1, PAX2, PAX3), which regulate nervous system patterning and regional fate; LIM homeodomain transcription factors (LHX1, LHX9, MNX1), which regulate cell fate and migration during development; the homeodomain transcription factors ONECUT1, ONECUT2, SIX1, SIM2, SHOX2, PITX1, ZIC1, ZIC2, and OTP; and the non-homeobox transcription factors TBX5 and FOXF1.

As in SPNs, phase D also elicited greatly elevated expression of *CDKN2A* (more than 40-fold, Fig. 7d) and *CDKN2B* (8-fold), which promote senescence and apoptosis in many contexts ^37-39^.

These results reveal shared principles for the transcriptional changes in striatal and cortical neurons in HD: phase C involves the continuous erosion of positive features of their respective identities, whereas phase D involves a later, more-rapid loss of negative features of neuronal identities.

### Neuronal loss (phase E) affects fast-expanding cell types

The highly variable levels of somatic CAG-repeat expansion among different types of pyramidal neurons (Fig. 2,3), the high repeat-length threshold for *HTT* toxicity in cortical neurons (~150 CAGs, Fig. 4), and our hypothesis that phase C/D transcriptional changes lead to neurodegeneration, together made a strong, testable prediction: across all types of pyramidal neurons, somatic expansion should have a strong negative relationship to cell survival in HD.

To estimate the survival rates of the various types of pyramidal neurons in HD, we sampled nuclei from 100 donors (50 persons with HD, 50 unaffected controls). We focused on two cortical areas – one that resembled most cortical areas in somatic expansion dynamics (anterior cingulate cortex, BA32) and another that had been the outlier (motor cortex, BA4). We analyzed these 200 samples by snRNA-seq, using the data to quantify loss of each type of neuron in each of the two cortical areas and 100 brain donors. For each type of pyramidal neuron, we estimated attrition in HD based on the relative numbers of such neurons in persons with HD compared to unaffected controls.

**Figure 8.**
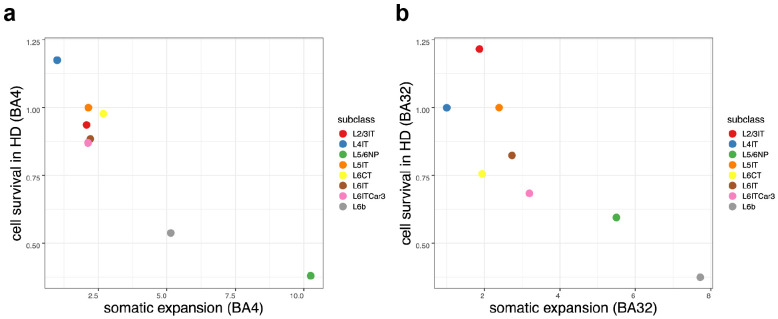
Loss of specific types of pyramidal neurons in HD, in BA4 (**a**) and BA32 (**b**), in relation to these pyramidal types' relative amounts of somatic expansion. Cell survival rates (y-axis) are calculated from the ratio of the group medians (median abundance in cortical samples among 50 persons with HD relative to median abundance among 50 unaffected control brain donors). Relative amounts of somatic expansion (x-axis) are estimated from regression of each neuron's CAG-repeat length against independent variables for donor and "subclass" (pyramidal neuron type).

In both cortical areas, pyramidal neurons' vulnerability to loss in HD was strongly related to their tendency to have expanded their CAG repeats somatically, with high-expansion types exhibiting much more loss than low-expansion types, and a clear relationship across the entire range (Fig. 8). L6b and L5/6NP neurons suffered particularly profound attrition, having declined by 40-60% in persons with HD relative to their abundance in unaffected controls (Fig. 8).

Thus, the various types of pyramidal neurons appear to be vulnerable to HD neurodegeneration to an extent predicted by somatic expansion of their *HTT* CAG repeats.

## Discussion

These results indicate that cortical neurons in HD traverse a sequence of pathogenic phases analogous to those traversed by striatal neurons – but do so with highly variable kinetics and distinct gene-expression sequelae. These phases involve decades of slow somatic expansion of the disease-causing CAG repeat tract (phase A); much-faster further expansion of this repeat (phase B); pervasive transcriptional changes that commence at about 150 CAGs, eroding the positive features of their respective identities (phase C); a de-repression crisis (phase D); and then elimination (phase E).

Our results nominate unifying explanations for the long-noted differences in the timing, severity, and gene-expression changes in HD pathologies in cortex vs. striatum. Differences in timing and severity appear to arise primarily from differing rates of somatic CAG-repeat expansion: only Layer 6b cortical pyramidal neurons appeared to acquire long somatic CAG-repeat expansions at almost SPN-like rates, with most pyramidal types exhibiting much less expansion. The long-noted differences between striatal and cortical gene-expression changes in HD result in part from a quite intriguing phenomenon. Upon reaching the high toxicity threshold of about 150 CAGs, cortical and striatal projection neurons commence almost completely different gene expression changes during phase C (Fig. 5c) – changes that are almost completely uncorrelated with each other and thus likely have different physiological sequelae and different downstream effects on other neurons and glia. However, the striatal and cortical phase C changes share a surprising property: the genes that decline in expression are overwhelmingly genes whose expression defines the identity of the affected neuronal type, with cortical neurons losing expression of their own, distinct identity-defining genes (Fig. 6). An important focus of future work will be to identify a cellular mechanism for such changes, and to explain how such distinct sets of phase C changes give way to a phase D that is quite similar between striatal and cortical neurons.

The possibility that corticostriatal projections are fundamental to mechanism in HD – with repeat-length-dependent pathology in one brain area eliciting secondary pathology in the other – has long intrigued neuroscience, leading to ideas that the pathophysiological mechanism might involve retrograde spreading of aggregation-driving prions ^14,15^, excitotoxicity ^9-11^, or loss of synaptic connections (corticostriatal disconnection) ^7,12^. We believe that our results suggest reconsidering this longstanding conception of HD pathology. First, our results suggest that the same fundamental dynamic propels degeneration of cortical and striatal neurons in HD – a dynamic that is cell-autonomous, in which expansion of each neuron's own CAG-repeat tract beyond ~150 CAGs is necessary and sufficient to initiate a pathological sequence of events. Second, we find that cortical pyramidal neurons of all types – regardless of molecular identity, connectivity, laminar location, or cortical area – are vulnerable to phase C pathology in those neurons whose own CAG-repeat tracts have expanded beyond the 150-CAG threshold (Fig. 5), and that pyramidal neurons suffer attrition in HD in a way that is strongly predicted by their somatic expansion rates (Fig. 8). The appearance of "resilience" or "vulnerability" in specific cortical cell types (or layers) thus arises from variation in their average rates of somatic CAG-repeat expansion, which determine whether a small or a large fraction of cells reach the toxicity threshold during a human lifetime.

Single-cell genomics has been creating ever-more-elaborate taxonomies of cortical pyramidal neurons based on their RNA expression patterns ^31,32^. However, the functional significance of these distinctions has been uncertain, as physiological differences among most of these neuronal types remain to be found ^35^. Here we identified an important functional difference that appears to underlie their vulnerability in HD: dramatic differences in their somatic DNA-repeat expansion. In fact, pyramidal neurons' fine-grained molecular identities appeared to be far more consequential for repeat expansion than their cortical areas or laminar locations were (Fig. 3).

All types of pyramidal neurons exhibited at least some somatic CAG-repeat tract expansion and underwent phase C transcriptional changes in those neurons in which the 150-CAG threshold was crossed (Fig. 4). However, specific types of pyramidal neurons (L6b, L5/6NP) exhibited particularly large amounts of somatic expansion (Fig. 2) and profound loss (Fig. 8) in persons with HD. The roles of L6b and L5/6NP neurons in intracortical and corticothalamic circuits will be worth considering in relationship to cognitive and psychiatric symptoms in HD, which often appear alongside motor signs and significantly impact quality of life and caregiver burden ^40,41^.

We propose that the apparent resilience (and vulnerability) of neuronal populations of various types comes not from connectivity, nor from differential sensitivity to HTT toxicity, but instead from whether they have low (or high) rates of somatic DNA-repeat expansion – which determines whether a small (or large) fraction of neurons reach the biologically consequential threshold of about 150 CAGs during a human lifetime. In principle this could also be generally true of other kinds of neurons throughout the brain, a possibility that will be interesting to test in future work.

Our results suggest that cortical neurons – even more than striatal neurons – may spend the longest period of their lives (perhaps 95-100%) with an innocuous *HTT* gene, then experience HTT toxicity in short, staggered temporal windows. Such a model suggests that in the cerebral cortex, as in the striatum, there might be great therapeutic leverage in slowing somatic DNA-repeat expansion as a way to prevent HD in persons who have inherited HD-causing alleles. Most importantly, these results suggest that a therapy that successfully slowed or stopped somatic DNA-repeat expansion would postpone or prevent neurodegeneration in the cerebral cortex as well as the striatum.

## Methods

### Experimental model and study participant details

We analyzed brain tissues from the Harvard Brain Tissue Resource Center/NIH NeuroBioBank (HBTRC/NBB), which were obtained by application (request #2496, "Neocortical cellular changes in Huntington’s Disease").

Brain donors were recruited by the HBTRC/NBB in a community-based manner across the USA. The HBTRC procedures for informed consent by the donor's legal next-of-kin and distribution of de-identified post-mortem tissue samples and demographic and clinical data for research purposes are approved by the Mass General Brigham Institutional Review Board. Post-mortem tissue collection followed the provisions of the United States Uniform Anatomical Gift Act of 2006 described in the California Health and Safety Code section 7150 and other applicable state and federal laws and regulations. Human brain tissue was obtained (for data generation) from the HBTRC/NBB (NBB request ID# 1835). Federal regulation 45 CFR 46 and associated guidance indicates that the generation of data from de-identified post-mortem specimens does not constitute human participant research that requires institutional review board review.

The HBTRC/NBB confirmed HD diagnosis and excluded clinical comorbidity and presence of unrelated pathological findings by reviewing medical records and by formal neuropathological assessment. The 1985 Vonsattel et al. grading of neostriatal pathology^42^ was used for diagnosis. Diagnosis on early cases is done using histological stainings and polyglutamine immunohistochemistry ^42-45^. Positivity in pontine gray neurons rules out HD-like-2 neuropathology ^46^, and cerebellar dentate neurons are mildly positive even in very early cases, while Purkinje cells are negative (unlike in cerebellar ataxia CAG expansion cases).

### Isolation of nuclei from brain tissue

Nuclei were isolated from frozen brain tissue using a density-gradient method ^30^. Briefly, frozen tissue was homogenized in Nuclei EZ Lysis Buffer (MilliporeSigma, #NUC101) supplemented with NxGen^®^ RNase Inhibitor (1 U/mL; Biosearch Technologies, #30281) using Dounce homogenizers. Homogenates were filtered through 70 μm cell strainers (Bel-Art, #H13680-0070) and centrifuged at 500 × g for 5 min at 4 °C. Supernatants were discarded and pellets were resuspended in 300 μL of G30 buffer (30% iodixanol, 3.4% sucrose, 20 mM tricine, 25 mM KCl, 5 mM MgCl_2_; pH 7.8). An additional 1 mL of G30 was gently underlaid to form a cushion, and the sample was layered above it. After centrifugation at 8,000 × g for 10 min at 4 °C, supernatants were removed. Nuclei pellets were washed once in 1 mL wash buffer (1% BSA in 1× PBS with 1 U/mL NxGen RNase Inhibitor), followed by 500 × g for 5 min at 4 °C. Nuclei were resuspended in 50 μL wash buffer and counted on a LUNA-FL Dual Fluorescence Cell Counter (Logos Biosystems).

### Preparation of snRNA-seq libraries

Nuclei encapsulation and library preparation were performed with Chromium Next GEM Single Cell 3′ Reagent Kits v3.1 (10X Genomics, PN-1000121) or Chromium GEM-X Single Cell 3′ Reagent Kits v4 (10X Genomics, PN-1000691) following the manufacturer’s protocol, with minor modifications during cDNA amplification (see Single-cell measurement of CAG-repeat length in snRNA-seq experiments below). Libraries were sequenced on Illumina NovaSeq 6000 or NovaSeq X platforms.

### Single-cell measurement of CAG-repeat length in snRNA-seq experiments

We previously developed an approach to simultaneously measure HTT CAG-repeat length and transcriptomes in individual nuclei ^30^. In brief, a whole-transcriptome library and an HTT-CAG targeted library were generated and linked by shared 10X cell barcodes.

During the cDNA amplification step (step 2.2a of the standard 3′ scRNA-seq protocol from 10X), we added two spike-in primers targeting the 5′ region upstream of the HTT CAG repeat to 1 μM each:

Spike-in A: 5′-CCCAGAGCCCCATTCATTGCC-3′

Spike-in B: 5′-GGCGACCCTGGAAAAGCTGATG-3′

In step 2.2d, the extension time was increased to 2 min. The amplified cDNA was split to generate (1) the conventional snRNA-seq library (10 μL) by continuing the standard 10X workflow and (2) an HTT-CAG library (4 μL) by targeted PCR using the UltraRun LongRange PCR Kit (QIAGEN, #206444; total reaction volume 20 μL) with following primers and PCR program:

Biotin IllumR-HTT-C:

5′-/5Bio/GTCTCGTGGGCTCGGAGATGTGTATAAGAGACAGCCTTCGAGTCCCTCAAGTCCTT C-3′

Illum_f_10X_3p_B:

5′-TCGTCGGCAGCGTCAGATGTGTATAAGAGACAGCTACACGACGCTCTTCCGATCT-3′

PCR program: 93 °C 3 min; 18-19 cycles of 93 °C 30 s, 60 °C 30 s, 68 °C 8 min; 72 °C 10 min; hold 4 °C.

The amplified HTT-CAG library was size-fractionated with SPRI beads (Beckman Coulter, #B23319) using 0.4X (Long, “L”) and 1.0X (Short, “S”) selections. DNA was eluted in 10 μL water. “L” and “S” fractions (either both or “L” only) were mixed with 10 μL of Dynabeads MyOne Streptavidin C1 (Thermo Fisher Scientific, #65002) pre-washed in 2X Wash & Binding buffer (2 M NaCl, 1 mM EDTA, 10 mM Tris-HCl, pH 7.5), incubated for 30 min at RT with rotation, washed 3 times in 1X Wash & Binding buffer, and resuspended in 10 μL water.

Bead-bound templates were then amplified for 12–14 cycles with unique pairs of standard Nextera i7/i5 indexing primers (the same index pair was used for “L” and “S” libraries when both were sequenced together) using the same kit and cycling conditions (total PCR volume 40 μL). PCR products were purified by 1.0X SPRI (eluted in 10 μL) and prepared for long-read sequencing.

For PacBio libraries, multiple samples were pooled equimolarly and prepared using SMRTbell Express Template Prep Kit 2.0 (Pacific Biosciences, #100-938-900) following PN 101-892-000 v01 (Jan 2020). “L” and “S” libraries were loaded on different flow cells and sequenced on Sequel IIe with a 100 pM loading concentration.

For Oxford Nanopore libraries, unpooled samples were barcoded and pooled with Native Barcoding Kit 24 V14 (ONT, SQK-NBD114.24; protocol V_NBA_9168_v114_revR_30Jan2025). The pooled libraries (35–50 fmol) were loaded on FLO-PRO114M flow cells and sequenced on PromethION 2 Integrated (P2i) instruments for 72 h with Super-accurate (SUP) base-calling.

### Mapping of snRNA-seq profiles to cortical cell taxonomies

We sought to classify each of the hundreds of thousands of neuronal gene-expression profiles (from snRNA-seq experiments) into multiple cortical taxonomies, including the Siletti taxonomy (the most detailed and current taxonomy, as of this work), and the Hodge/Tasic taxonomy (to which data on axonal projections and laminar locations have been mapped by earlier studies).

In this project, we generated 10x snRNA-seq data in either village or single donor experiments. To all of these nuclei, we applied an scPred model that classifies each nucleus as one of 7 main cell types as well as the MapMyCells algorithm that assigns each nucleus to a subcluster_name / cluster_name / supercluster_name (see the Siletti taxonomy).

To analyze glutamatergic neurons, we selected the nuclei that were classified as "glutamatergic" by scPred (with the assignment probability max.prob > 0.8) and also were assigned by MapMyCells to one of 4 excitatory neurons' superclusters (in the Siletti taxonomy).

For these glutamatergic neurons, we further ran the scPred glutamatergic and GABAergic subclass / subtype models that were trained on the data from Hodge et. al

### Mapping MapMyCells cluster_name to glutamatergic subclasses

To map each MapMyCells "cluster_name" (which in the Siletti taxonomy are children of 4 excitatory neuron superclusters ) to scPred's glutamatergic subclasses, we used the village data from BA4 and (separately) BA32 to build confusion matrices between "cluster_name" and glutamatergic subclass assignments. For each cluster_name with at least 10 neurons in it, we computed subclass frequencies for the neurons labelled with this cluster_name and then assigned this cluster_name to the highest frequency subclass. We determined that for each of the cluster_names more than 60% (most often, more than 90%) of all the neurons belonged to a single glutamatergic subclass, to which we then associated it. We performed this mapping independently for the BA4 and BA32 data and confirmed that the mapping results derived from these two analyses/regions were identical.

### Negative binomial regression analyses of somatic CAG-repeat expansion

For a cell c with an HD-causing allele length measurement, we define its phase A somatic expansion value, phaseA_expansion, as the difference between its CAG-repeat length (capped at 100 CAGs) in c and the inherited CAG-repeat length for the donor from whom cell c was sampled. In more formal terms,

phaseA_expansion=max(0,min(100,CAG_LENGTH)-INHERITED_CAG_LENGTH)

The reason for capping CAG_LENGTH at 100 is that at about this CAG-repeat length there is a transition from phase A to a far-faster phase B, and the CAG-repeat expansion dynamic accelerates significantly, and could (we don't know) be governed by different cell-type-specific dynamics. So we focus on phase A expansion kinetics.

We model the phase A CAG-repeat expansion using a Negative binomial regression of this form:

phaseA_expansion(c)∼NB(μ(c),v)

where the log mean of this negative binomial model, log(μ), is a linear function L of the following cell level covariates for a cell c:

L=donor+region+subcluster

***region*** is the brain region (cortical area) from which cell c was sampled. ***donor*** is the donor from whom cell c was sampled. Donor-level effects implicitly include any effects of age, genetic background (e.g. modifiers), and disease progression. ***subcluster*** is the subcluster (in the Siletti taxonomy) to which cell c was assigned.

Fig. 3 reports values of the *region* and *subcluster* coefficients, for subclusters with a threshold number of measurements.

### Negative binomial regression analyses of gene-expression changes

In order to identify genes whose expression changes as the HD-causing *HTT* allele's CAG-repeat tract expands, in our recent study we fitted per-gene Negative Binomial Regression models that used various covariate selections as well as their functional forms ^30^.

In the current study, we adopted the same approach to modeling gene expression as a function of CAG-repeat length (CAG_LENGTH) and additional covariates, with a slight modification of the covariates used in the current model. The NBR models for gene expression in the cortex are as follows:

$$E_{g}(c)∼NB(\mu_{g}(c),v_{g})$$

where the log mean of this negative binomial model, $\log(\mu_{g})$, is a linear function L of the following cell level covariates for a cell c:

$$L=log(N)+f_{m,n}(CAG\_LENGTH)+phaseD.prob_{S,M}+donor+region+subcluster$$

N is the total count of UMIs (RNA transcripts from any gene) in cell c

$f_{m,n}(\mathrm{CAG}\_\mathrm{LENGTH})$ is a function family (parameterized by positive integers m and n *such that* m<n) of the CAG-repeat length of the HD allele in cell c. f can be a simple function of CAG_LENGTH (for example, it can be CAG_LENGTH itself), or it can be defined, for example, as a “hinge function” (in which CAG_LENGTH has no effect until a threshold m is reached, then has a continuously escalating effect with further increase in CAG_LENGTH):

$$f_{m,n}(CAG\_LENGTH_{c})=0,\text{if}\;CAG\_LENGTH<m;\;\;\text{min}(CAG\_LENGTH,n)\;\text{-}\;m,\text{if}\;CAG\_LENGTH>=m$$

$\mathrm{phase}D.{\mathrm{prob}}_{S,M}$ is a covariate used to measure the probability of cell c having entered phase D. It is modeled as the following logistic function of the total normalized count of the phaseD gene transcripts (computed as the sum of the UMI counts normalized to 100k over all 59 phaseD genes):

$$phaseD.prob_{S,M}(\#phaseD\_UMIs)=\frac{1}{1+e^{-S\;*\;(\#phaseD\_UMIs-M)}}$$

where S and M are the steepness and the midpoint of the function respectively. ***region*** is the brain region (cortical area) from which cell c was sampled. ***donor*** is the donor from whom cell c was sampled. Donor-level effects implicitly include any effects of age, genetic background (e.g. modifiers), and disease progression. ***subcluster*** is the subcluster (in the Siletti taxonomy) to which cell c was assigned.

The R function we used to fit the per-gene Negative Binomial Regression models is very slow when run on a data set of this size. Preliminary analyses revealed that essentially all of the highly significant results were arising from the small fraction of cells with repeat-tracts longer than 150. To make the recognition of phase C and phase D genes more computationally tractable as the data set expanded, we downsampled (by 90%) the data in the interval of (35-100] CAG repeats, while retaining all the cells with CAG-repeat length > 100. All NBR models were thus fitted to data from 9,140 pyramidal neurons, of which 5,477 (or 60%) had CAG-repeat length <= 100 and 3,663 (or 40%) had CAG-repeat length > 100.

The statistical significance of the f(CAG_LENGTH) and phaseD coefficients was used to recognize (respectively) phase C and phase D genes, which were used (respectively) for Fig. 4-6 and Fig. 7. To reduce noise in calculating the phase C median fold-change for C+ (phase C expression-increasing) and C- (phase C expression-decreasing) gene sets, we imposed an additional inclusion threshold that a gene must have been detected in 90% of the cells in the analysis.

### Analyses of cell loss

For snRNA-seq analysis of pyramidal neuron loss across 100 donors, we processed sets (“villages”) of 20 brain donors (including HD and control donors) en bloc as single pooled samples, in a manner we previously described ^30,47^. Each 20-donor village was pooled and handled together from nuclei extraction through sequencing, and each village was partitioned across eight encapsulation reactions. Donor-of-origin for each nucleus was assigned computationally from combinations of transcribed SNPs detected in the nucleus’s RNA-seq reads ^48^.

We first normalize the abundance of each pyramidal neuron type relative to that of layer 5 intra-telencephalic-projecting (L5IT) neurons, a high-abundance pyramidal population that undergoes just modest amounts of somatic expansion. We found quantification in this manner to yield estimates that were more consistent (less variable) across unaffected control donors – superior in this regard to simply quantifying abundance as a fraction of all nuclei sampled – potentially because numbers of certain other populations (especially OPCs and interneurons) change with age.

These quantifications were made in each donor individually. For each pyramidal type, we then calculated the ratio of (median abundance among persons with HD) to (median abundance among unaffected donors).

## Supplementary Material

1
