# Supplementary material for: A shared DNA-repeat toxicity threshold, reached somatically at cell-type-specific rates, unites cortical and striatal neurodegeneration in Huntington’s disease
Source: bioRxiv. 2025 Dec 11:2025.12.09.688862. Preprint. [Version 1] doi: 10.64898/2025.12.09.688862 (PMC12713137; doi:10.64898/2025.12.09.688862)

bioRxiv preprint doi: https://doi.org/10.64898/2025.12.09.688862; this version posted December 11, 2025. The copyright holder for this preprint (which was not certified by peer review) is the author/funder, who has granted bioRxiv a license to display the preprint in perpetuity. It is made available under aCC-BY 4.0 International license.

Supplementary Materials

Supplementary Table 1. Cortical areas analyzed by snRNA-seq.

Area	Name
**BA4**	Primary Motor Cortex (precentral gyrus)
**BA32**	Dorsal Anterior Cingulate Cortex
**BA46**	Dorsolateral Prefrontal Cortex (dlPFC)
**BA17**	Primary Visual Cortex (V1)
**BA19**	Associative Visual Cortex (primarily V3)
**BA37**	Fusiform Gyrus (higher associative visual)
**BA41**	Primary Auditory Cortex (A1)
**BA42**	Associative Auditory Cortex (A2)
**BA22**	Middle Temporal Gyrus (higher order auditory)
**BA3**	Primary Somatosensory Cortex (S1)
**BA40**	Associative Somatosensory Cortex (S2)
**BA7**	Superior Parietal Gyrus (multisensory associative)

bioRxiv preprint doi: https://doi.org/10.64898/2025.12.09.688862; this version posted December 11, 2025. The copyright holder for this preprint (which was not certified by peer review) is the author/funder, who has granted bioRxiv a license to display the preprint in perpetuity. It is made available under aCC-BY 4.0 International license.

Supplementary Figure 1. Allele-specificity of somatic CAG-repeat instability in the cerebral cortex, in seven brain donors. Unlike other figures in this work, which focus on the somatically-expanding, HD-causing *HTT* allele, this figure shows both alleles, to visualize the relative stability of each donor's shorter CAG-repeat tract.

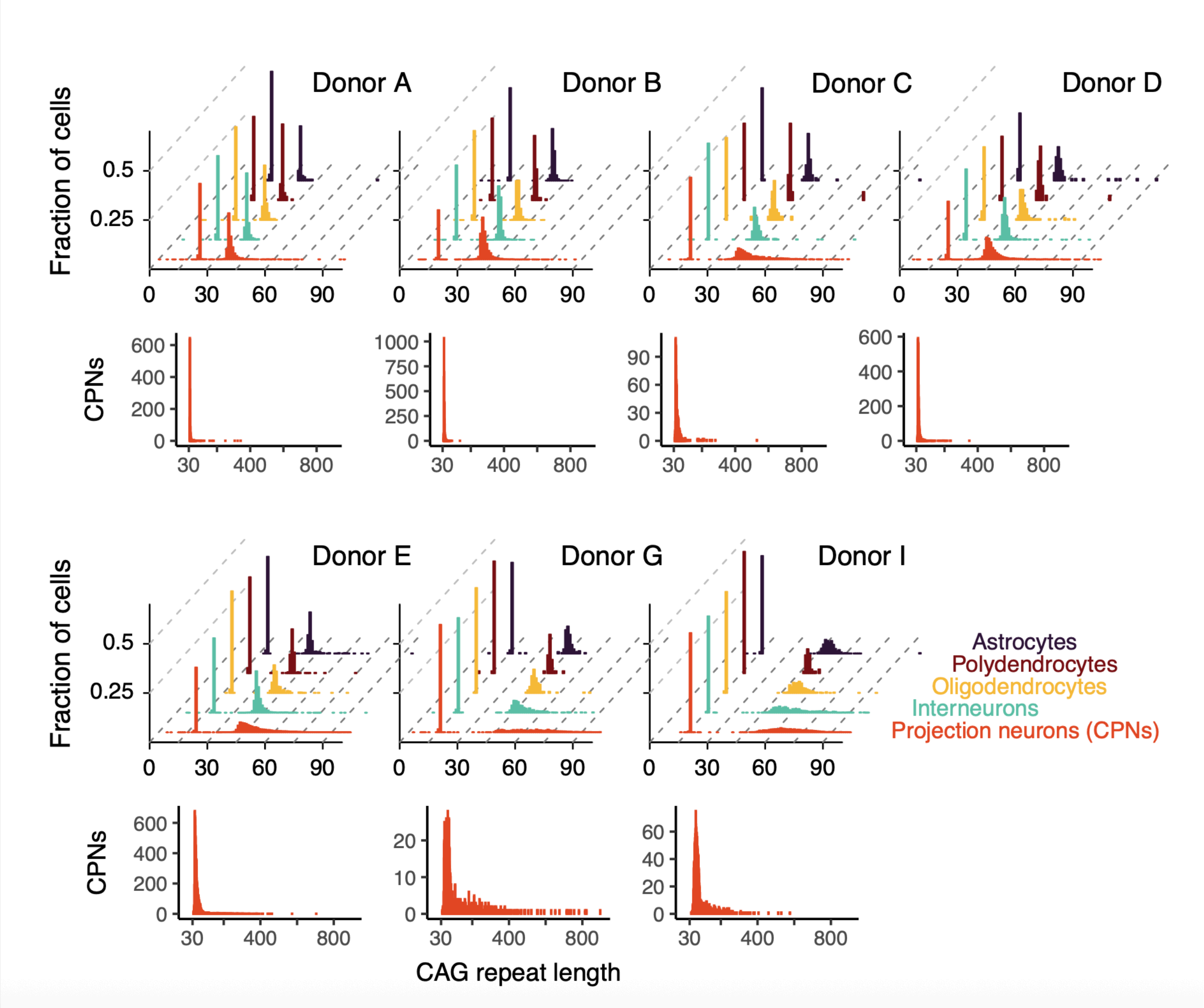

bioRxiv preprint doi: https://doi.org/10.64898/2025.12.09.688862; this version posted December 11, 2025. The copyright holder for this preprint (which was not certified by peer review) is the author/funder, who has granted bioRxiv a license to display the preprint in perpetuity. It is made available under aCC-BY 4.0 International license.

Supplementary Figure 2. Somatic CAG-repeat expansion in specific types of cortical GABAergic neurons, in multiple brain donors with HD, here shown as histograms of neuron counts. Each row/color corresponds to a different type of GABAergic neurons. LAMP5+ interneurons consistently show the most somatic expansion, though very few of them expand their CAG-repeat tracts beyond 100 CAGs.

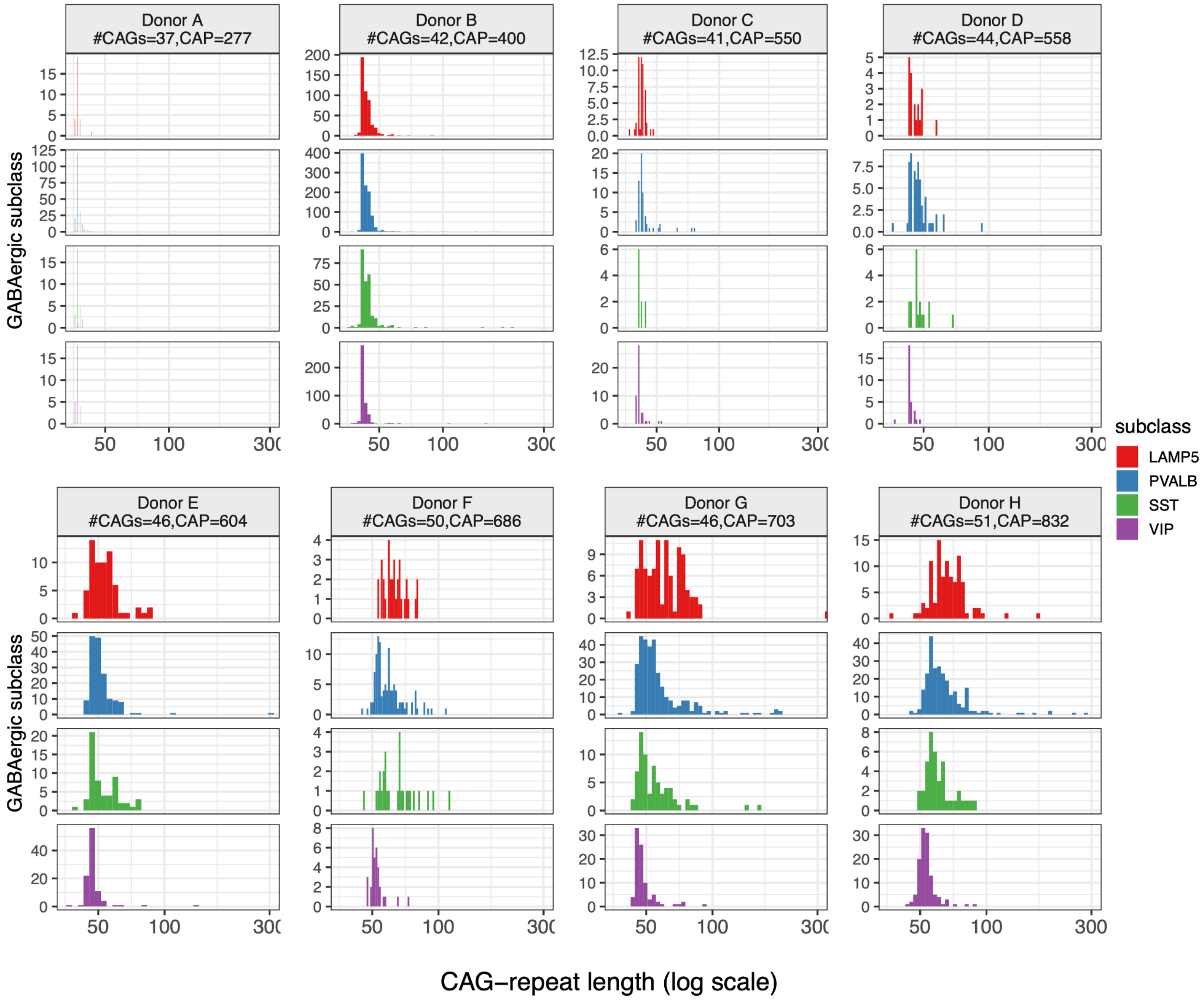

bioRxiv preprint doi: https://doi.org/10.64898/2025.12.09.688862; this version posted December 11, 2025. The copyright holder for this preprint (which was not certified by peer review) is the author/funder, who has granted bioRxiv a license to display the preprint in perpetuity. It is made available under aCC-BY 4.0 International license.

Supplementary Figure 3. Replication (across brain donors) of estimates of the relative amounts of somatic *HTT* CAG-repeat expansion in different pyramidal neuron subtypes. Each circle corresponds to a pyramidal neuron subtype (from the high-resolution Siletti taxonomy) for which we have quantified somatic CAG-repeat expansion. These 20+ pyramidal subtypes are each indicated with a circle; the colors of the circles reflect supertypes (L2/3IT, etc.) from the earlier, lower-resolution (but more extensively annotated) Hodge taxonomy. On the y-axes are estimates from a deeply sampled brain donor from whose cerebral cortex we sampled ten cortical areas. On the x-axes are estimates from a group of other brain donors, focusing on estimates from motor cortex (BA4, left plot) or anterior cingulate cortex (BA32, right plot). Note that, for L6b and L5/6NP pyramidal subtypes (gray and green circles), loss of the neurons with the most somatic expansion during the course of HD (see **Fig. 8**) would cause us to under-estimate their somatic expansion, by an amount that varies from donor to donor; this may be why these gray and green points diverge from the otherwise-linear replication relationship formed by the other pyramidal subtypes. Also note that, because some donors have more somatic expansion than others (due e.g. to age and genetic modifiers), the cardinal values of these estimates are donor-specific (it is their *relative* values that appear to persist across donors).

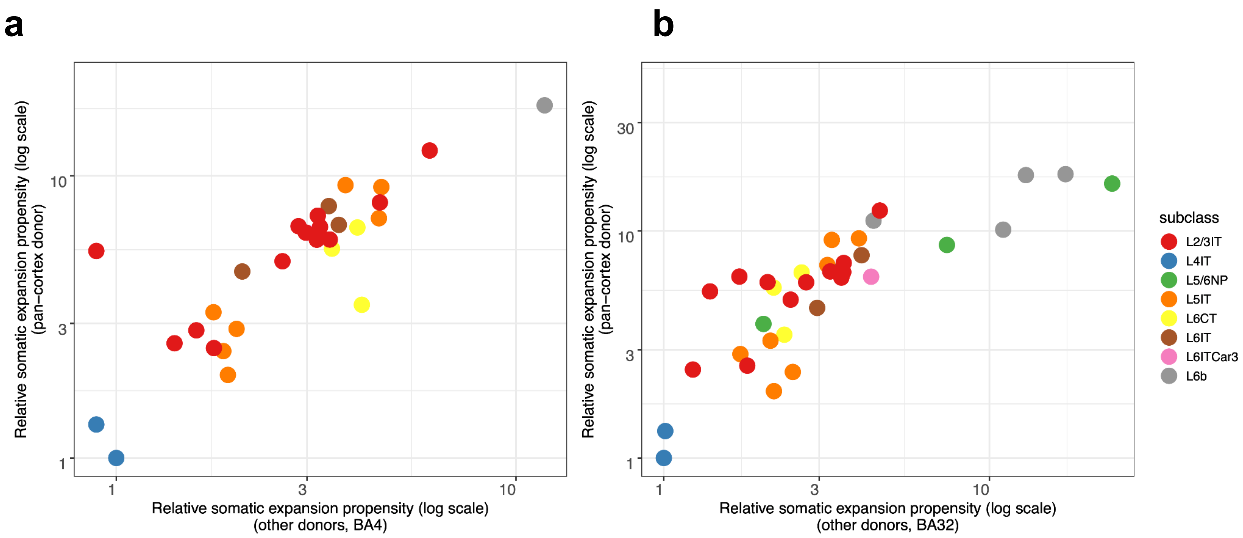

bioRxiv preprint doi: https://doi.org/10.64898/2025.12.09.688862; this version posted December 11, 2025. The copyright holder for this preprint (which was not certified by peer review) is the author/funder, who has granted bioRxiv a license to display the preprint in perpetuity. It is made available under aCC-BY 4.0 International license.

Supplementary Figure 4. Somatic CAG-repeat expansion in subtypes of pyramidal neurons, here shown as count histograms and focusing on the HD-causing allele. (**a**) Subtypes of layer 2/3 intratelencephalic-projecting (L2/3IT) neurons; (**b**) Subtypes of layer 5/6 near-projecting (L5/6NP) neurons.

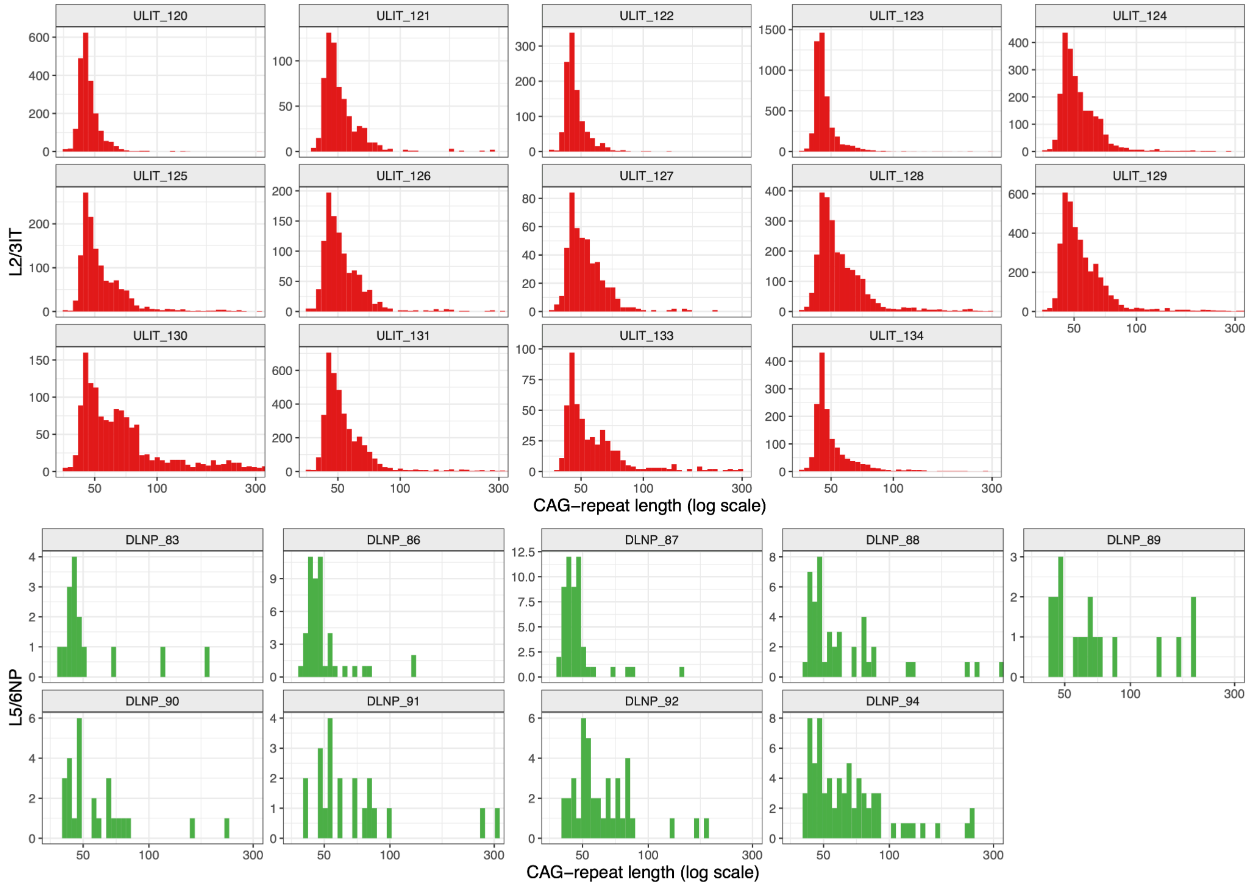

bioRxiv preprint doi: https://doi.org/10.64898/2025.12.09.688862; this version posted December 11, 2025. The copyright holder for this preprint (which was not certified by peer review) is the author/funder, who has granted bioRxiv a license to display the preprint in perpetuity. It is made available under aCC-BY 4.0 International license.

Supplementary Figure 5. Relative rates of somatic *HTT* CAG-repeat expansion across pyramidal neuron subtypes, in relationship to expression levels of a variety of genes known or proposed to regulate the stability of the *HTT* CAG repeat.

Rigorous statistical inference of these relationships is challenging, since cell types have a complex, structured, and partially hierarchical set of similarity/dissimilarity relationships – they cannot be considered independent samples from a space of cell-type possibilities, and p-values from statistical tests that do not incorporate these complex relationships are thus almost certainly misleading.

It is important to remember that individual genes tend to change in expression in the context of large gene-expression programs that involve concerted changes in the expression of very many other genes – often hundreds of genes – together. Thus, expression measurements for a single gene are also indirect proxies for many other genes' expression levels, and for unmeasured cellular properties (such as neuronal activity patterns or metabolic activities) that also potentially affect somatic instability and DNA maintenance pathways. In this context, one should be reluctant to attribute causality to individual genes based on observational/correlational analyses like those below.

The analysis does make it possible, though, to place a low upper bound on the extent to which nuclear RNA expression level of any one of these factors explains the variation (up to 20-fold) in pyramidal cell types' somatic CAG-repeat expansion.

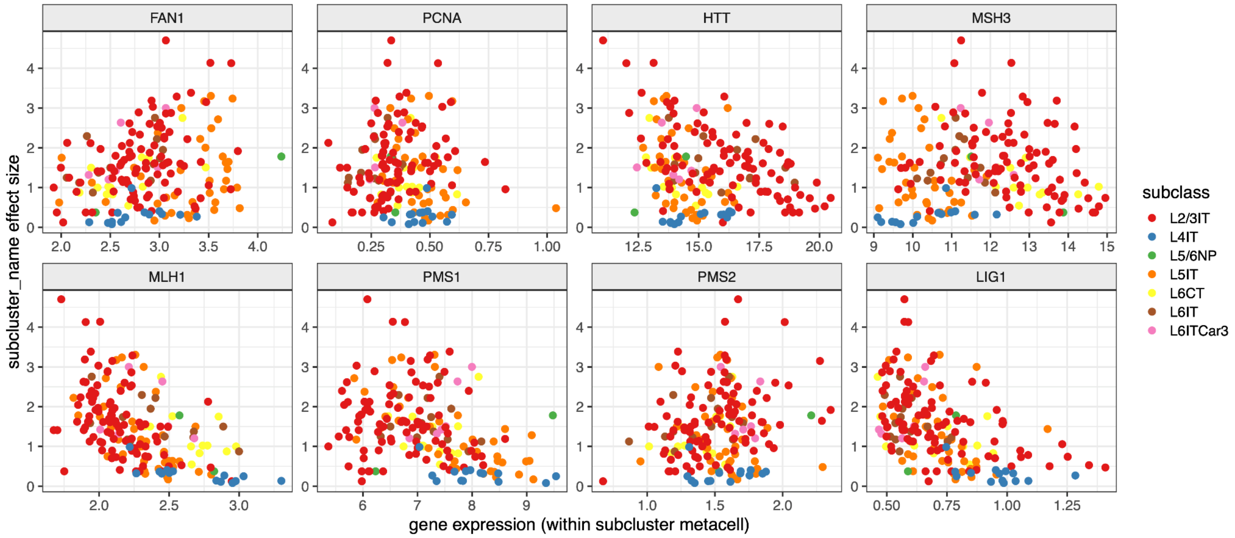

bioRxiv preprint doi: https://doi.org/10.64898/2025.12.09.688862; this version posted December 11, 2025. The copyright holder for this preprint (which was not certified by peer review) is the author/funder, who has granted bioRxiv a license to display the preprint in perpetuity. It is made available under aCC-BY 4.0 International license.

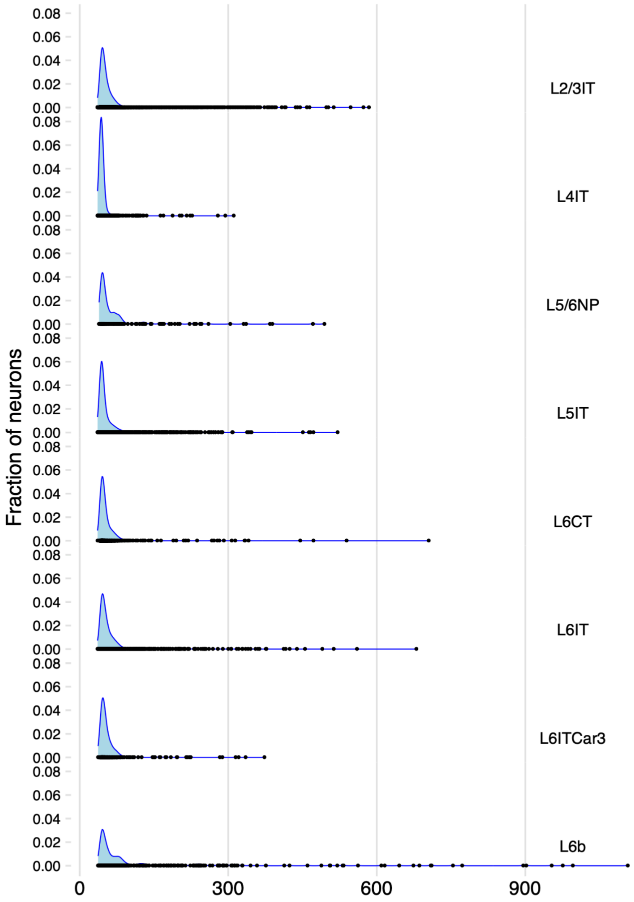

Supplementary Figure 6. Distributions of CAG-repeat lengths in eight types of cortical glutamatergic (pyramidal) neurons from the same person with HD. Blue shaded areas are smoothed density estimates of the repeat-length distribution. Overplotted black points show the CAG-repeat length measurements in individual glutamatergic neurons. In all eight glutamatergic neuron subclasses, the repeat-length distribution exhibits an armadillo-like shape, in which the DNA-repeat tract in most neurons has undergone modest expansion (up to about 90 CAGs) but in a small fraction of neurons it has undergone far greater expansion (up to 500+ CAGs).

bioRxiv preprint doi: https://doi.org/10.64898/2025.12.09.688862; this version posted December 11, 2025. The copyright holder for this preprint (which was not certified by peer review) is the author/funder, who has granted bioRxiv a license to display the preprint in perpetuity. It is made available under aCC-BY 4.0 International license.

Supplementary Figure 7. Phase C gene-expression changes arise in cortical neurons with somatic CAG-repeat expansion beyond 150 CAGs. Analyses of motor cortex (**a**) and anterior cingulate cortex (**b**) in four additional persons with HD. On each pair of panels, each cortical glutamatergic neuron is represented by both a blue point (lower plot) and an orange point (upper plot): blue points show the median fold-change of a set of 115 genes that decrease in expression with CAG-repeat expansion (C- genes); orange points show the median fold-change of a set of 142 genes that increase in expression with CAG-repeat expansion (C+ genes). (**a, above**) Motor cortex (BA4). (**b, below**) Anterior cingulate cortex (BA32).

a

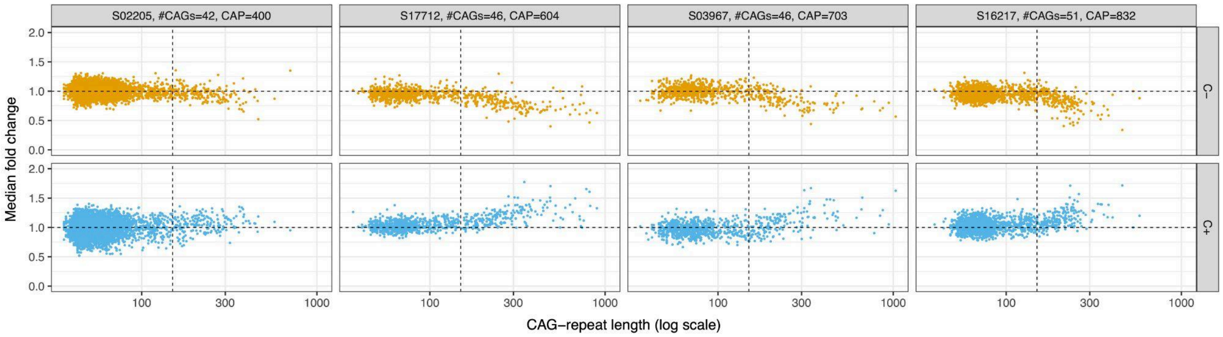

b

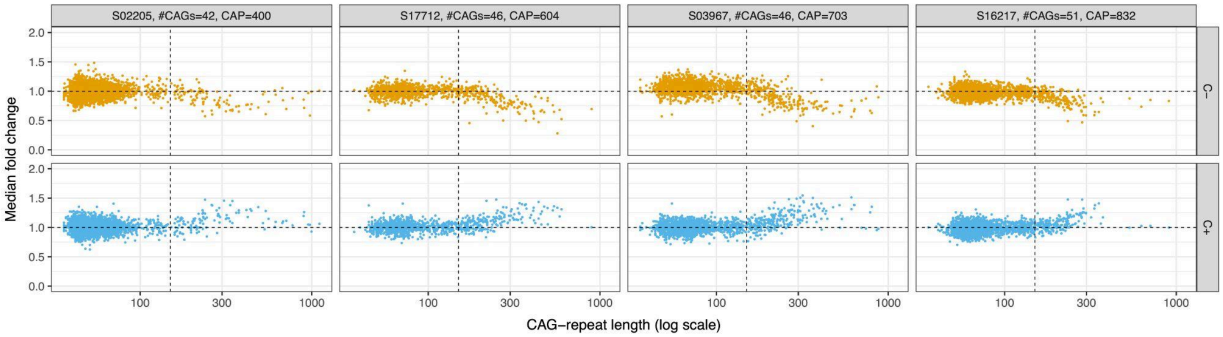

bioRxiv preprint doi: https://doi.org/10.64898/2025.12.09.688862; this version posted December 11, 2025. The copyright holder for this preprint (which was not certified by peer review) is the author/funder, who has granted bioRxiv a license to display the preprint in perpetuity. It is made available under aCC-BY 4.0 International license.

Supplementary Figure 8. Phase C gene-expression changes arise in those pyramidal neurons – of all types – with somatic CAG-repeat expansion beyond 150 CAGs. Each panel shows data for a specific type of pyramidal neuron from the Hodge taxonomy. On each panel, each neuron is represented by both a blue point and an orange point: orange points show the median fold-change of a set of 115 genes that decrease in expression with CAG-repeat expansion (C- genes); blue points show the median fold-change of a set of 142 genes that increase in expression with CAG-repeat expansion (C+ genes).

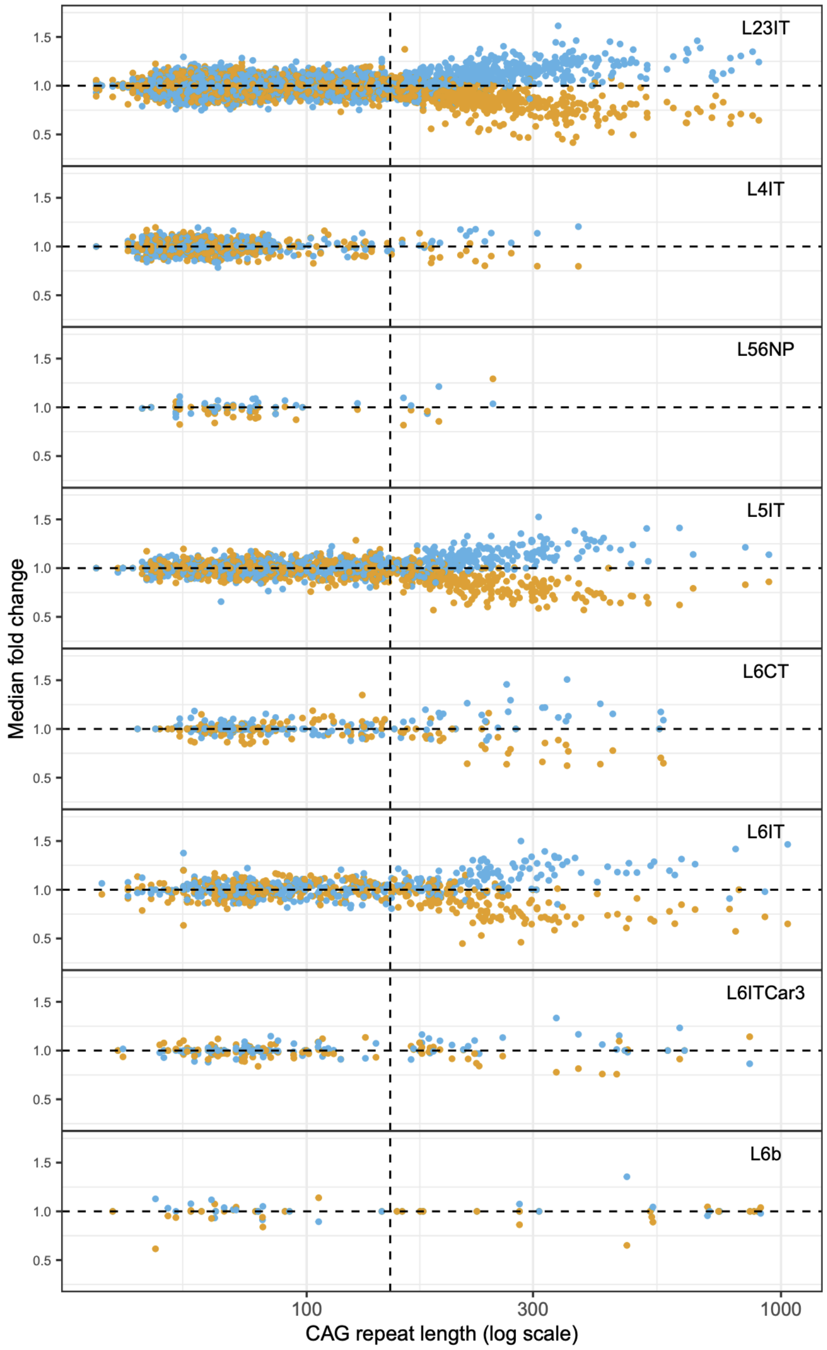

bioRxiv preprint doi: https://doi.org/10.64898/2025.12.09.688862; this version posted December 11, 2025. The copyright holder for this preprint (which was not certified by peer review) is the author/funder, who has granted bioRxiv a license to display the preprint in perpetuity. It is made available under aCC-BY 4.0 International license.

Supplementary Figure 9. Phase D gene-expression changes in additional brain donors (here shown all together), in two cortical areas (left, motor cortex BA4; right, anterior cingulate cortex BA32). Points represent individual neuronal nuclei. y-axis: de-repression score, the number of transcripts (UMIs) detected from 59 phase D genes that are normally silent in cortical projection neurons.

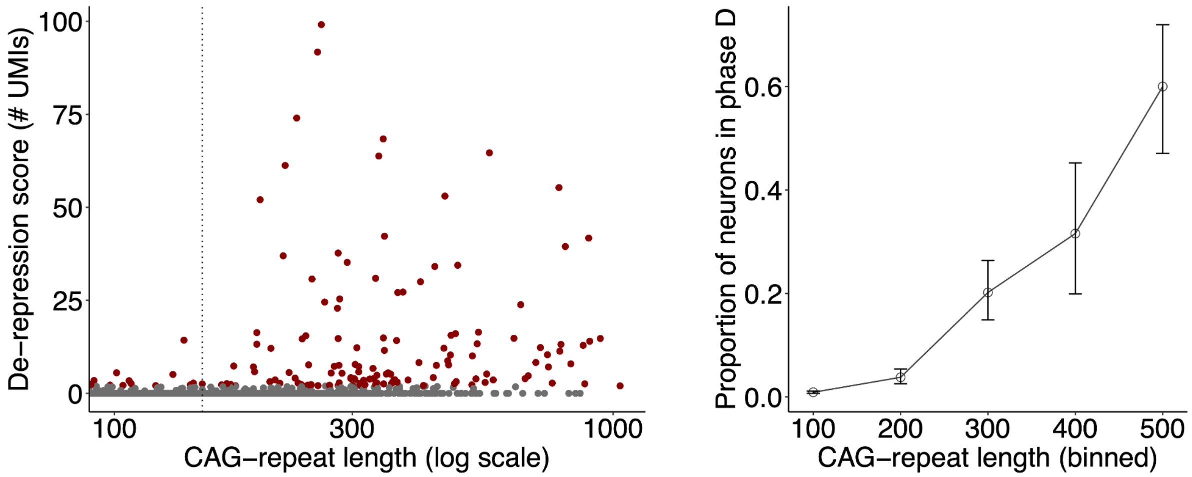

Supplement: 1 [file NIHPP2025.12.09.688862v1-supplement-1.pdf]
